# Modification of Pronated Foot Posture after a Program of Therapeutic Exercises

**DOI:** 10.3390/ijerph17228406

**Published:** 2020-11-13

**Authors:** Raquel Sánchez-Rodríguez, Sandra Valle-Estévez, Peñas Albas Fraile-García, Alfonso Martínez-Nova, Beatriz Gómez-Martín, Elena Escamilla-Martínez

**Affiliations:** 1 Nursing Department, Podiatric Clinic of University of Extremadura CPUEX, 10600 Plasencia, Spain; rsanrod@unex.es (R.S.-R.); bgm@unex.es (B.G.-M.); escaelen@unex.es (E.E.-M.); 2 Podiatric Clinic Las Lomas, Avda Salinera 6L-6K, 19005 Guadalajara, Spain; sandravalle10@gmail.com; 3 Physiotherapy Clinic PAFG, Tenerías 4, 10610 Cabezuela del Valle, Spain; palfragar@yahoo.es

**Keywords:** foot posture, pronated foot, health, therapeutic exercise, foot core, flat foot

## Abstract

Working on the intrinsic musculature of the foot has been shown to be effective in controlling pronation. However, the potential coadjuvant effect that involving other muscle groups might have on foot posture remains unknown. The aim was, therefore, to assess whether a 9-week intrinsic and extrinsic foot and core muscle strength program influenced foot posture in pronated subjects. The participants were 36 healthy adults with pronated feet that were randomly assigned to two groups. The experimental group (*n* = 18) performed a strengthening exercise protocol for 9 weeks (two sessions of 40 min per week), while the control group (*n* = 18) did not do these exercises. After 9 weeks, the foot posture index (FPI) scores of the two groups were analyzed to detect possible changes. The FPI at the baseline was 8.0 ± 1.5. After the 9 weeks, the experimental group showed significantly reduced FPI from 8.1 ± 1.7 to 6.4 ± 2.1 (*p* = 0.001), while the control group had the same score as pre-intervention (FPI 8 ± 1.2, *p* = 1.0). The FPI scores showed no significant differences by sex. Strengthening of the intrinsic and extrinsic foot and core muscles contributed to improving foot posture in adults, reducing their FPI by 1.66 points.

## 1. Introduction

Foot pronation is a natural movement, necessary for the normal development of gait, since it contributes to the absorption of the ground reaction forces [1,2]. However, overpronation causes the collapse of the medial longitudinal arch of the foot, thus causing more tension in certain muscles of the foot and leg, i.e., posterior tibialis [3]. Pronated feet present rearfoot eversion and dorsiflexion with abduction of the forefoot in a static position, and in addition, have a strong relationship with the medial longitudinal arch (MLA). It is suggested that maintaining the integrity of the MLA can prevent numerous musculoskeletal injuries [4].

Pronated foot posture is common in adulthood, with a prevalence of around 21% [5], and it may be related to deformities of the first ray of the foot, such as the hallux valgus [6,7]. Furthermore, this biomechanical alteration can also affect other areas, such as the knee [8]. Thus, pronation influences the time and intensity of lumbopelvic muscle activation, giving rise to low back pain or other dysfunctions at that level [9].

There are several methods to treat the pronated foot, such as custom foot orthoses, external modifications of the footwear, exercises to strength the muscles involved or even surgery [10,11,12,13]. Strength exercises are a highly recommended method in the treatment of childhood flat foot, since muscle enhancement plays a very important role in the development of the lower limbs in the growth of the child. The lack of treatment can often lead to short- and long-term problems with deformities in adolescence and adulthood [13].

In adults, exercising the intrinsic foot musculature (short-foot exercises) has been shown to be effective in reducing the height of the internal arch or pronation in healthy subjects and sportspersons [14,15]. Recently Pabón-Carrasco et al. [16] demonstrated that foot posture could be modified with shot foot exercises, with a decrease in navicular drop measurements.

Additionally, stabilization of the trunk while executing movements of the entire lower limb can directly affect the foot [17,18]. Thus, therapeutic exercise of the extrinsic foot and core musculature could enhance this effect of reducing the pronated posture. If this is indeed so, it would be a good alternative to include in rehabilitation plans for pronated feet, although currently, its potential effect is still unknown. The objective of this study was to establish a protocol of exercises for the short and extrinsic foot and core muscles, lasting 9 weeks, and to evaluate the possible modification of the foot posture in adult subjects with pronated feet.

## 2. Materials and Methods

### 2.1. Participants

The study was conducted between March–June of 2019. The sample comprised 36 adult subjects (15 men and 21 women) with pronated feet. Their mean age was 22.6 ± 4.4 years, BMI of 23.6 ± 2.9 kg/m^2^, and mean foot posture index (FPI) of 8 ± 1.5 (range, from 6 to 11). The anthropometric of the whole group, by gender, is presented in Table 1, with the male group being heavier, taller and with a higher body mass index (*p* < 0.05). The inclusion criteria were: (a) presenting pronated feet, and (b) age between 18 and 40 years. The exclusion criteria were subjects who (a) had treatment with plantar supports, (b) had previous surgery, and (d) were carrying out any type of exercise for therapeutic purposes. The study was conducted in accordance with the Declaration of Helsinki, and the protocol was approved by the Bioethics Committee of the University of Extremadura (date; 2012–2018), who gave a positive report on the research project and for the clinical trial (Id: 105/2018). All participants signed their informed consent.

Individuals were randomly assigned (using STATA v12 statistical software: College Station, TX: Stata Corp LP. USA) to two groups, one experimental (*n* = 18, 7 men and 11 women, mean age 23.6 ± 5.9 years and BMI 23.2 ± 3.2 kg/m^2^) who would carry out the exercise protocol, and another control (*n* = 18, 8 men and 10 women, mean age 21.6 ± 1.9 years and BMI 23.9 ± 2.6 kg/m^2^) who would continue with their daily activities. There were no significant differences in age or BMI between groups, p = 0.182 and 0.452, respectively.

### 2.2. Instruments and Procedure

Two experienced podiatrists measured the foot posture index. The inter-observer reliability (intraclass correlation coefficient (ICC)) was calculated between both measurements, showing a value of 0.871, that could be considered as a good level of reliability and consistent with other research [19].

For these measurements, the subjects were asked to stand in their relaxed stance position with double limb support, their arms relaxed at their sides and looking straight ahead. The six criteria used in the FPI were talar head palpation, supra and infra malleolar curvature, calcaneal frontal plane position, prominence in the region of the talonavicular joint, congruence of the medial longitudinal arch, and abduction/adduction of the forefoot on the rearfoot. Each criterion was scored on a scale ranging between − and +2, with the foot considered to be neutral when the score was between 0 to 5, and pronated from 6 to +12 [20]. Due to the good reliability between both podiatrists, the mean of the two measurements was used for the analysis. In order to preserve the independence of the data, the left foot FPI was used for statistical analysis [21].

**Figure 1:**
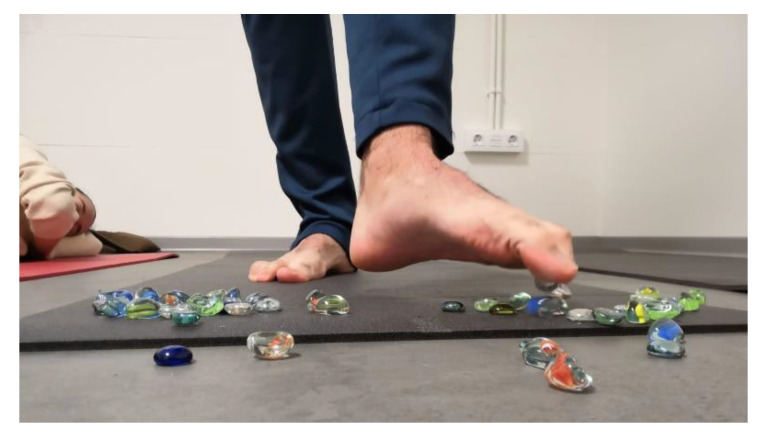
Picking up small glass stones using toes and depositing the stones elsewhere.

**Figure 2:**
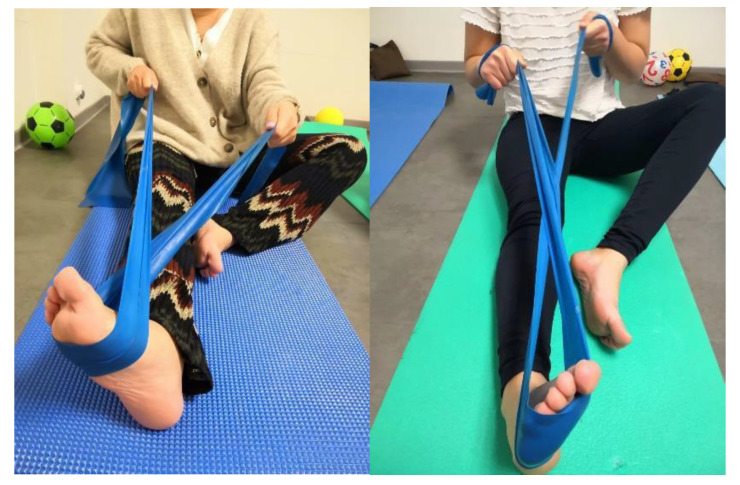
Inversion and eversion resistive movement with an elastic band.

**Figure 3:**
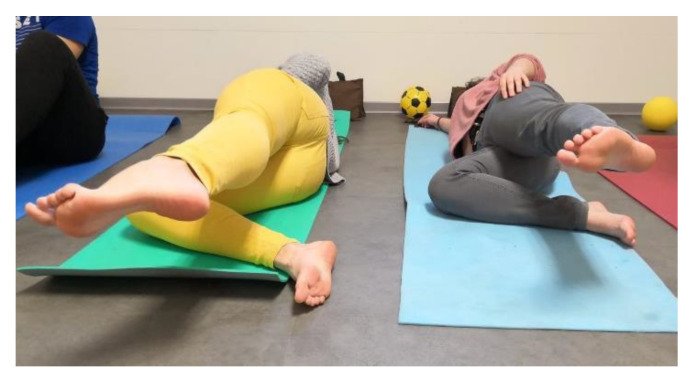
Hip abduction.

Foot exercise protocols of 4 to 6 weeks in length were shown to be effective [15,16], so, we argue that extending this period could provide better hyper pronation recovery. Additionally, including lower limbs and core exercises in the protocol could help to provide a better muscle control of the leg and probably improve the over-pronation [22]. All 18 subjects in the control group underwent a supervised protocol for foot, lower limb and core exercises for 9 weeks. This protocol was designed by an experimented physiotherapist (author 3). Each session comprised 11 active-resisted exercises:

(1) Walking on their heel and forefoot. In upright position, subjects must walk (a) with the sole support of their heel and (b) with the support of metatarsal heads and phalanges. Feet must be aligned with the legs.
(2) Walking on medial and lateral border of the foot. Subjects must walk with the sole support of (a) medial longitudinal arch and (b) lateral longitudinal arch.
(3) Picking up small objects with the toes. In standing position subjects must pick up little stones with the toes, and release them in other places (Figure 1).

(4) Resistive inversion and eversion with an elastic band. While seated with a straight leg, subjects must move the foot in eversion and inversion with the resistance of an elastic band (Figure 2).

(5) Hip abduction. In the lateral position, the leg in contact with the ground is bent. The contralateral leg will be aligned with the trunk. Subjects must contract 8 s moving away the leg from the other, and 8 s of muscle relaxation when lowering it again (Figure 3).

(6) Strength of the erector spinae. With the subject in the prone position, extended upper limbs in prolongation of the body. Subjects must perform a slight elevation of the trunk. A total of 8 s of contraction and 8 s of muscle relaxation.
(7) Strength of the abdomen. With the subject in the supine position, and knees flexed, slightly separated and aligned with the feet, hip elevation. On the abdomen there will be a weights 2 kg, and a ball, which they have to stabilize with the pelvis.
(8) Strength of the obliques of the abdomen. With the subject in the supine position and knees flexed, slightly separated and aligned with the feet, flexed trunk the right arm must touch the left knee, left arm and right knee, respectively. A total of 8 s of contraction and 8 s of muscle relaxation.
(9) Ball in the legs. With the subject in the supine position, with flexed knees and a ball between the legs contracting the adductors (8 s of contraction and 8 s of muscle relaxation).
(10) Balance on an unstable base. In the standing position subjects must move in eversion and inversion, supporting in the toes and also the heel.
(11) Balance on an unstable base and destabilization. In the standing position a couple of subjects must maintain balance bouncing a ball between them.

The focus was to work on the long flexor of the hallux, long and short flexors of the other toes, abductor hallucis, lumbricals, interossei, fibularis longus and fibularis brevis, tibialis posterior and tibialis anterior, gastrocnemius, hamstrings, hip adductors, iliopsoas, gluteus medius, erector spinae, external obliques of the abdomen, transversus abdominis, and rectus abdominis. The duration of each exercise was 1:30 min per limb and 3 min for the lumbopelvic muscles. A total of 18 sessions of 40 min were performed. All subjects completed all sessions.

### 2.3. Statistical Analysis

In order to assess the normal distribution of the sample, a Shapiro–Wilk test was performed. All values of foot posture presented a significance >0.05, so data were distributed normally and parametric tests were applied. The FPI data were subjected to descriptive analysis using the Student’s *t*-test for paired samples (pre-post) and the Student’s *t*-test for independent samples (men–women, experimental–control). Chi-squared tests pre- and post-intervention were used to compare the distributions of the two groups’ FPI data. The calculations were performed using the SPSS software version 15.0 for Windows (IBM, Armonk, NY, USA). The significance level was set at 5% (*p* < 0.05).

## 3. Results

The overall FPI (both groups) at baseline was 8.0 ± 1.5 (men 8.0 ± 1.5, women 8.1 ± 1.5). By group, the experimental group started from an FPI of 8.1 ± 1.7, which, after the 9 weeks, was significantly reduced to 6.4 ± 2.1 (*p* = 0.001). In the control group, the initial FPI was 8.0 ± 1.2, and this remained at identical values after the 9 weeks. Table 2 presents the differences by sex, with no significant differences being found in either group.

The initial FPI of the experimental group showed 15 subjects to have pronated feet and three to have highly pronated feet. After completing the exercise program (final FPI), there were six subjects with neutral feet, 11 with pronated feet, and one highly pronated (Table 3). There was no modification of the FPI in the control group, and there was now a significant (*p* = 0.001) difference in the distribution of the two groups.

## 4. Discussion

The combination of lower limb muscle and core strengthening exercises proved effective in improving the foot posture in adult subjects with pronation. While other work [15] has observed changes in individual parameters (calcaneal inversion/eversion), the overall FPI parameter was only reduced by one point and the difference compared to the initial measurements was not significant. With the present conjoint muscle strengthening protocol, we achieved a decrease of 1.66 points in the FPI, with a migration of eight subjects (two of which were highly pronated, along with six pronated subjects) to more neutral postures. While other studies have previously focused on strengthening the short plantar musculature [16]—considered a useful tool in dealing with pathologies whose etiology includes excessive pronation of the foot—our results show that working with the extrinsic and core musculature could also provide great benefits in the neuromuscular pattern of the lower limb. Thus, powerful hip external rotators would be able to slow down the internal rotation of the lower limb, avoiding knee valgus [23], and therefore, pronation. The core musculature would reinforce lumbopelvic stability, allowing for correct execution of the lower limbs’ movements [17,22,24].

In this way, a rehabilitation or treatment plan for pronated feet (whether symptomatic or not) should target together all of the musculature that is, in one way or another, involved in the control of body stability so as to obtain the maximum possible results at a distal level. This muscle strengthening could reduce the prevalence of pronation related injuries, increase the flexibility and range of joint movement in the lower limb and lower back [25], and improve balance [15,17,22]. Given the beneficial effects deriving from it, this specific muscle exercising should not be forgotten, even if the patient is receiving orthopedic treatment. To achieve correct rehabilitation of the pronated foot, it is important that these therapeutic exercises be carried out under professional supervision and with individualized adaptation.

Although the literature indicates that women tend to present slightly more pronated feet than men [26], in our sample the improvement in foot posture did not seem to be related to sex, with the exercises being equally effective in both sexes.

### Limitations of the Study

The present study has some limitations: (1) This is a short-term study, (2) it is unknown whether the effect obtained with muscle strengthening had a short (beyond the 9 weeks), medium, or long term duration. Until having long-term data to see if these differences persist for 6 or 12 months following the training program, the results could be considered interesting but not consistent, as the potential effect of exercises could disappear if they are not continued. This variable should therefore be taken into account in future research. Until then, these exercises should continue to be performed twice a week to maintain adequate muscle tone and to preserve the optimal results found after the 9 weeks of exercise. One strength of this work is that, to the best knowledge of the authors, there has not been such a comprehensive foot, leg and core exercise protocol for the treatment of pronated feet. As a result of this research, we intend to continue working in this field, expanding the sample of the population under study, extending the period of intervention and evaluating the long-term effects of physical exercise on pronated feet.

## 5. Conclusions

Performing an intrinsic and extrinsic foot and core muscle strengthening exercise protocol for 9 weeks improved the hyper pronation in this sample of adults with pronated feet, resulting in the foot posture becoming closer to neutrality, regardless of sex. Based on our results, we suggest the inclusion of these muscle strengthening exercises in all pronated foot treatment plans, even when the subject is treated with foot orthoses.

## Figures and Tables

**Table 1:** Anthropometric data of the sample by gender.

	Gender	*n*	Mean	SD	*p*
Weight	Men	15	77.6	7.5	<0.001
	Women	21	58.7	9.8	
Height	Men	15	175.1	6.3	<0.001
	Women	21	161.1	6.4	
BMI	Men	15	25.3	1.9	0.002
	Women	21	22.3	2.9	
Years	Men	15	24.3	6.3	0.099
	Women	21	21.3	1.6	

*t*-test for independent samples.

**Table 2:** Differences by gender in both groups.

	Men		Women	*p*
FPI Experimental	**PRE**	8.3 ± 1.7	8 ±1.8	0.887
	**POST**	7 ± 2.3	6.1 ± 2.1	0.457
FPI	**PRE**	7.7 ± 1.4	8.2 ± 1.1	0.430
Control	**POST**	7.7 ± 1.4	8.2 ± 1.1	0.430

*t*-test for independent samples.

**Table 3:** Changes in foot posture index (FPI) group after the 9 weeks of the physical activity.

	Neutral	Pronated	Highly Pronated	TOTAL
FPI Pre	0	15	3	18
FPI Post	6	11	1	18
*p*	0.001			

chi-square test.
